# Pretransplant spleen volume and outcome after hematopoietic stem cell transplantation (HSCT) in patients with acute myeloid leukemia (AML)

**DOI:** 10.1007/s00277-023-05353-9

**Published:** 2023-07-10

**Authors:** Alexander Pohlmann, Eva Bentgens, Christoph Schülke, David Kuron, Christian Reicherts, Julia Marx, Linus Angenendt, Jan-Henrik Mikesch, Georg Lenz, Matthias Stelljes, Christoph Schliemann

**Affiliations:** 1 Department of Medicine A, University Hospital Münster, Münster, Germany; 2 Department of Clinical Radiology, University Hospital Münster, Münster, Germany; 3 Department of Medicine II, University Hospital Schleswig-Holstein, 24105 Kiel, Germany

**Keywords:** Spleen volume, Hematopoietic stem cell transplantation, Acute myeloid leukemia, Engraftment, Non-relapse mortality

## Abstract

Allogeneic hematopoietic stem cell transplantation (HSCT) is an effective treatment modality for patients with acute myeloid leukemia (AML). Here, we investigated the predictive value of spleen volume on outcome parameters and engraftment kinetics after HSCT in a large cohort of AML patients. A total of 402 patients who received their first HSCT between January 2012 and March 2019 were included in this retrospective study. Spleen volume was correlated to clinical outcome and engraftment kinetics. Median follow-up was 33.7 months (95% confidence interval [CI], 28.9–37.4 months). Patients were subdivided based on median spleen volume of 238.0 cm^3^ (range 55.7–2693.5 cm^3^) into a small spleen volume (SSV) and a large spleen volume (LSV) group. LSV was associated with inferior overall survival (OS) after HSCT (55.7% vs. 66.6% at 2 years; *P* = 0.009) and higher cumulative incidence of NRM (28.8% vs. 20.2% at 2 years; *P* = 0.048). The adjusted hazard ratio for NRM in the LSV group was 1.55 (95% CI, 1.03–2.34). Time to neutrophil or platelet engraftment and the occurrence of acute or chronic graft-versus-host disease (GVHD) were not significantly different between both groups. Higher spleen volume at the time of HSCT was independently linked to adverse outcomes such as inferior OS and higher cumulative incidence of NRM in AML patients after HSCT. Engraftment kinetics and GVHD were not associated with spleen volume.

**Supplementary information:**

The online version contains supplementary material available at 10.1007/s00277-023-05353-9.

## Introduction

Advances in allogeneic hematopoietic stem cell transplantation (HSCT) and associated supportive care have resulted in increased survival rates of patients with acute myeloid leukemia (AML) [1]. However, HSCT procedure can result in severe treatment-related complications, mainly immunosuppression-related infections and graft-versus-host disease (GVHD) [1–3]. Rates of non-relapse mortality (NRM) have significantly decreased over the last decades [1, 3–6]. Horan et al. [6] reported that NRM at 3 years after allogeneic HSCT for AML patients in first complete remission decreased from 29% in the 1980s to a level of 15% between 2000 and 2004, although NRM attributed to infections remains a significant risk [6]. Thus, identification of risk factors for NRM remains relevant.

In patients with myelofibrosis undergoing allogeneic HSCT, splenomegaly has been linked to primary graft failure and poor outcome [7–9]. However, little is known on the association of spleen volume and outcome after HSCT in patients with AML. A recent study [9] showed that an enlarged spleen (greater than 320 cm^3^) was associated with poor neutrophil and platelet engraftment and inferior overall survival (OS) following HSCT. Similarly, Akpek et al. [10] reported that an enlarged spleen was associated with poor engraftment after HSCT in patients with myeloproliferative disorders and myelodysplastic syndrome; however, they found no difference in OS between groups of different spleen volumes. While the leading hypothesis is that an enlarged spleen in AML patients who receive HCST is indicative of poor clinical outcome, it remains controversial whether spleen volume is independently associated with outcome in those patients.

Here, we retrospectively investigated the association between pretransplant spleen volume and OS, cumulative incidence of relapse (CIR), NRM and hematological recovery in a large cohort of AML patients who underwent HSCT.

## Methods

### Study patients

**Fig. 1:**
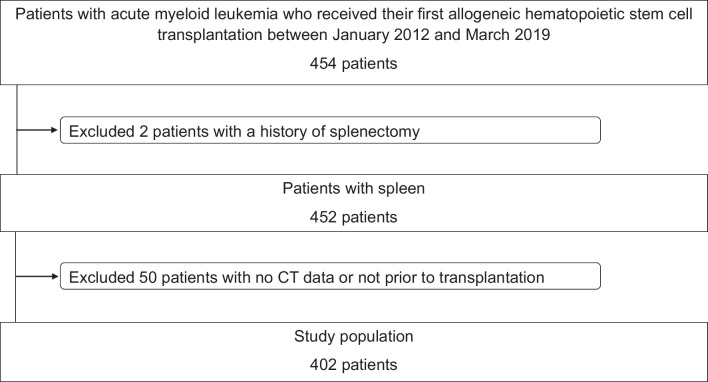
Patient selection flow

We retrospectively screened 454 patients with AML who received their first HSCT at the Bone Marrow Transplantation Center of the University Hospital Münster, Germany, between January 2012 and March 2019 (Fig. 1). Two patients were excluded from the study because they had a history of splenectomy and 50 patients were excluded as no pretransplant abdominal computer tomography (CT) scan was available for analysis. Thus, 402 patients were included in the final analysis. None of the patients had splenic vein thrombosis.

Patients in complete remission (CR) received a reduced intensity conditioning regimen consisting of fludarabine and a total body irradiation (4 × 2 Gy) or fludarabine in combination with treosulfan or busulfan. The non-myeloablative conditioning regimens largely consisted of fludarabine and a total body irradiation (2 × 2 Gy). Patients with active disease at the time of transplantation were treated with a high-dose melphalan-based sequential conditioning regimen, followed by fludarabine and a total body irradiation (4 × 2 Gy) or a combination of melphalan, fludarabine, and treosulfan.

Peripheral blood stem cells (PBSC) or bone marrow (BM) was obtained from healthy volunteers and infused on day 0 without further manipulation. Neutrophil engraftment was defined as the first of three consecutive days with at least 500 neutrophils/µl in the blood. Platelet engraftment was defined as platelet count over 20,000/µl in the blood without transfusion. Acute and chronic GVHD were assessed according to the standard criteria [11–14].

### Measurement of spleen volume using pretransplant CT scans

**Fig. 2:**
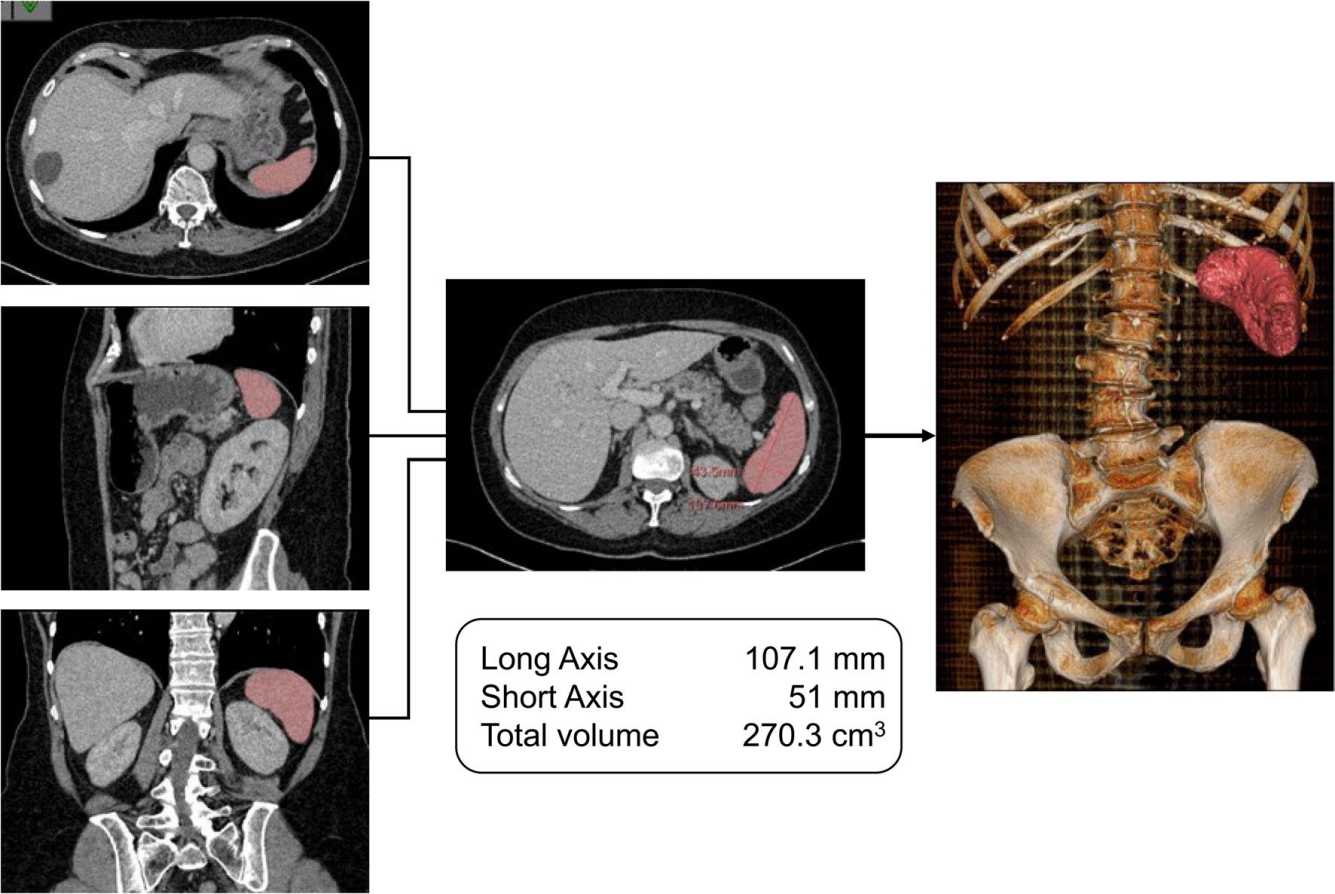
Measurement of spleen volume — case presentation. Presented is the spleen of a 60-year-old female patient who underwent allogeneic hematopoietic stem cell transplantation on 29th June 2012. The induction therapy included cytarabine and daunorubicin (7 + 3 scheme) and cytarabine and mitoxantrone (HAM scheme). Subsequently, the disease was in first complete remission. As the conditioning therapy, this patient received a reduced intensity regime with fludarabine (cumulative 150 mg/m^2^) und busulfane (cumulative 6.4 mg/kg of body weight). Neutrophil engraftment was achieved on day 15 after transplantation and platelet engraftment on day 12. She had a good recovery after transplantation and no relapse. On 13th December 2017 she died from a candida sepsis. In this study, the patient was part of the large spleen volume (LSV) group

For each patient the spleen volume was determined with the CT scan data obtained within 30 days prior to HSCT. CT examinations were done using dual source 64- and 128-slice CT scanners (Somatom® Definition and Definition Flash, Siemens AG, Medical Solutions, Forchheim, Germany). All CT data sets were reconstructed with a slice thickness of 1.5 mm and a reconstruction increment of 0.6 mm in axial orientation. Analysis of splenic volume and attenuation was performed using IntelliSpace Portal version 11.1 (Philips Medical Systems Nederland B.V., Best, The Netherlands) by means of a semiautomatic threshold-based contour-finding algorithm. Spleen contours were drawn based on density differences with surrounding tissues. Computer-generated organ boundaries were manually corrected by an examiner, who was blinded to the clinical status of the patients. To optimize measurements, images were scanned in a fan-like approach in three planes: the coronal, sagittal, and transversal plane (Fig. 2).

### Statistical analysis

Patient data were retrospectively retrieved from patient records. The cohort was divided into two groups (small spleen volume (SSV) and large spleen volume (LSV)) using the median spleen volume as the cut-off. The Mann–Whitney U test was used to compare continuous variables in both groups and chi-square test was used to compare categorial variables. Survival estimates were calculated using the Kaplan–Meier method and survival curves were compared using the log-rank test. CIR and NRM were calculated with the Aalen-Johansen estimator. All survival probabilities were calculated at 2 years. Median follow-up was calculated by the reverse Kaplan–Meier method. OS was defined as the time from the date of transplantation to the date of death by any cause. NRM was defined as the time from date of transplantation to the date of death for patients without AML relapse. CIR was defined as the time from date of transplantation to the date of relapse. Relapse-free survival (RFS) was defined as the time from date of transplantation to the date of relapse or death by any cause, whichever came first. Primary cause of death was assessed according to previously published definition [15].

A multivariable Cox regression model was constructed to assess the significance of spleen volume as a prognostic factor with respect to OS and a multivariable Fine-Gray regression model was used to assess the significance of spleen volume as a prognostic factor with respect to CIR and NRM, adjusting for the variables age, sex, weight, body length, AML type, human leukocyte antigen (HLA) match, donor type, remission status, and cytomegalovirus (CMV) risk. The consistency of association with outcomes across subgroups was analyzed with test for interaction. Missing data were not imputed. *P*-values < 0.05 were considered statistically significant. Analyses were performed using RStudio version 1.1.463 (RStudio®, USA) and SPSS software version 26 (IBM®, USA).

## Results

### Baseline characteristics

A total of 402 AML patients were eligible for inclusion. Median spleen volume was 238.0 cm^3^ (range 55.7–2693.5 cm^3^). Median length of the longest axis was 103.8 mm (range 59.9–203.9 mm) and of the shortest axis 56.9 mm (range 27.5–124.4 mm). The median spleen volume was used to dichotomize the total cohort into 201 patients with small spleen volume (SSV, spleen volume lower than or equal to 238.0 cm^3^) and 201 patients with large spleen volume (LSV, spleen volume higher than 238.0 cm^3^). Baseline patient characteristics according to spleen volume are shown in Table 1. The median age was 58 years. Prior to transplant, 202 patients (50.2%) were in complete remission (CR) and 200 patients (49.8%) had active AML. PBSC was applied to 389 (96.8%) patients; 11 patients (2.7%) received a BM graft. Anti-thymocyte globulin (ATG) was given to 319 of 402 patients (79.4%). Cyclosporine in combination with methotrexate as GVHD prophylaxis was applied to 177 patients (44.0%); 221 patients (55.0%) were given cyclosporine in combination with mycophenolate mofetil. Ninety-nine patients (24.6%) received granulocyte colony-stimulating factor (G-CSF): 48 patients in SSV group and 51 patients in LSV group (*P* = 0.729). As expected, there were more male patients in the LSV group (69.7% vs. 48.3%, *P* < 0.0001). Median body length was 172 cm in the SSV (range 153.00–196.00 cm) and 176 cm in the LSV group (range 149.00–198.00 cm) (*P* < 0.0001). There was no significant association between type of AML (secondary AML vs. de novo) and spleen volume (*P* = 0.120). Furthermore, all patients in SSV and LSV group were Epstein-Barr virus (EBV) IgG (immunoglobulin G) positive.

**Table 1:** Patient characteristics

Variables	Small spleen volume SSV	Large spleen volume LSV	Total	*P*-value
***N***	201	201	402	
Age, years				0.301
Median (range)	58 (18–76)	58 (18–74)	58 (18–76)	
Sex, no (%)				< 0.0001
Male	97 (48.3)	140 (69.7)	237 (59.0)	
Female	104 (51.7)	61 (30.3)	165 (41.0)	
AML type, no (%)				0.120
De novo	156 (77.6)	144 (71.6)	300 (74.6)	
t AML	13 (6.5)	9 (4.5)	22 (5.5)	
s AML	32 (15.9)	48 (23.9)	80 (19.9)	
FAB classification, no (%)				0.229
M0	20 (10.0)	14 (7.0)	34 (8.5)	
M1	27 (13.4)	32 (15.9)	59 (14.7)	
M2	48 (23.9)	40 (19.9)	88 (21.9)	
M3	0	1 (0.5)	1 (0.2)	
M4	37 (18.4)	37 (18.4)	74 (18.4)	
M5	35 (17.4)	29 (14.4)	64 (15.9)	
M6	6 (3.0)	7 (3.5)	13 (3.2)	
M7	2 (1.0)	0	2 (0.5)	
Unknown	26 (12.9)	41 (3.5)	67 (16.7)	
ELN 2010, no (%)				0.366
Favorable	31 (15.4)	24 (12.0)	55 (13.7)	
Intermediate	114 (56.7)	116 (57.7)	230 (57.2)	
Adverse	56 (27.9)	61 (30.3)	117 (29.1)	
Remission status at Tx, no (%)				0.364
Complete remission	105 (52.2)	97 (48.3)	202 (50.2)	
CR1	84 (41.8)	82 (40.8)	166 (41.3)	
CR ≥ 2	16 (8.0)	14 (7.0)	30 (7.4)	
CRI	5 (2.5)	1 (0.5)	6 (1.5)	
Other	3 (1.5)	5 (2.4)	8 (2.0)	
Active AML	96 (47.8)	104 (51.7)	200 (49.8)	
Refractory	62 (30.8)	79 (39.3)	141 (35.1)	
Untreated relapse	31 (15.4)	20 (10.0)	51 (12.7)	
Donor age, years				0.915
Median (range)	36 (18–73)	36 (18–71)	36 (18–73)	
Donor type, no (%)				0.685
Related Mismatched	0	2 (1.0)	2 (0.5)	
Related Matched	60 (29.9)	51 (25.4)	111 (27.6)	
Unrelated Matched	108 (53.7)	117 (58.2)	225 (56.0)	
Unrelated Mismatched	30 (14.9)	26 (12.9)	56 (13.9)	
Haploidentical	3 (1.5)	5 (2.5)	8 (2.0)	
Sex mismatch, no (%)				0.890
Female donor/male recipient	30 (14.9)	31 (15.4)	61 (15.2)	
Other	171 (85.1)	170 (84.6)	341 (84.8)	
Graft source, no (%)				0.770
PBSC	192 (95.5)	197 (98.0)	389 (96.8)	
BM	7 (3.5)	4 (2.0)	11 (2.7)	
Unknown	2 (1.0)	0 (0)	2 (0.5)	
CMV risk, no (%)				0.486
D − /R-	50 (24.9)	49 (24.4)	99 (24.6)	
D + /R-	22 (10.9)	24 (11.9)	46 (11.4)	
D − /R +	37 (18.4)	48 (23.9)	85 (21.2)	
D + /R +	92 (45.8)	80 (39.8)	172 (42.8)	
Conditioning, no (%)				0.074
MAC	2 (1.0)	2 (1.0)	4 (1.0)	
RIC	103 (51.2)	83 (41.3)	186 (46.2)	
NMA	4 (2.0)	8 (4.0)	12 (3.0)	
SEQ	92 (45.8)	108 (53.7)	200 (49.8)	
Total body irradiation, no (%)				0.214
No TBI	108 (53.7)	97 (48.3)	205 (51.0)	
< 8 Gy	4 (2.0)	8 (4.0)	12 (3.0)	
≥ 8 Gy	89 (44.3)	96 (47.7)	185 (46.0)	
ATG, no (%)				0.712
ATG	158 (78.6)	161 (80.1)	319 (79.4)	
No ATG	43 (21.4)	40 (19.9)	83 (20.6)	
Immunosuppression, no (%)				0.038
CsA/MMF	100 (49.8)	121 (60.2)	221 (55.0)	
CsA/MTX	99 (49.2)	78 (38.8)	177 (44.0)	
Other	2 (1.0)	2 (1.0)	4 (1.0)	
Acute GVHD (until day + 100), no (%)				0.454
Grade 0–1	164 (81.6)	158 (78.6)	322 (80.1)	
Grade ≥ 2	37 (18.4)	43 (21.4)	80 (19.9)	
Chronic GVHD, no (%)				0.339
No cGVHD	123 (61.2)	132 (65.7)	255 (63.4)	
Mild	16 (8.0)	18 (9.0)	34 (8.5)	
Moderate	33 (16.4)	24 (11.9)	57 (14.2)	
Severe	29 (14.4)	27 (13.4)	56 (13.9)	
Primary cause of death, no (%)				0.519
Relapse	21 (30.0)	18 (19.1)	39 (23.8)	
Graft-Failure	3 (4.3)	3 (3.2)	6 (3.7)	
aGVHD	0 (0.0)	4 (4.3)	4 (2.4)	
cGVHD	9 (12.9)	15 (16.0)	24 (14.6)	
Infection	23 (32.9)	39 (41.5)	62 (37.8)	
Organ toxicity	7 (10.0)	5 (5.3)	12 (7.3)	
Other	4 (5.6)	6 (6.3)	10 (6.1)	
Unknown	3 (4.3)	4 (4.3)	7 (4.3)	
Body length, cm				< 0.0001
Median (range)	172.00 (153.00–196.00)	176.00 (149.00–198.00)	174.00 (149.00–198.00)	
CD 34 cell number, 10^6^ per kg body weight				0.580
Median (range)	7.2 (1.23–12.53)	7.4 (0.66–17.9)	7.3 (0.66–17.9)	
CD 45 cell number, 10^8^ per kg body weight				0.833
Median (range)	9.5 (0.5–36.8)	9.2 (2.0–45.9)	9.3 (0.5–45.9)	
CD 3 cell number, 10^8^ per kg body weight				0.432
Median (range)	2.6 (0.01–306.0)	2.5 (0.03–427.00)	2.5 (0.01–427.00)	
Neutrophil engraftment, no (%)				1.000
< 42 days	190 (94.5)	187 (93.0)	376 (93.5)	
> 42 days	0	0	0 (0.0)	
Not engrafted	11 (5.5)	14 (7.0)	26 (6.5)	
Platelet engraftment, no (%)				1.000
< 100 days	185 (92.0)	178 (88.6)	364 (90.5)	
> 100 days	0	0	0 (0.0)	
Not engrafted	16 (8.0)	23 (11.4)	38 (9.5)	
G-CSF stimulation, no (%)				0.729
Stimulation	48 (23.9)	51 (25.4)	99 (24.6)	
No stimulation	153 (76.1)	150 (74.6)	303 (75.4)	
Spleen volume				< 0.0001
Median (range)	157.9 (55.7–237.9)	327.3 (238.1–2693.5)	238.0 (55.7–2693.5)	
Spleen — short axis				< 0.0001
Median (range)	49.9 (27.5–86.5)	66.9 (37.2–124.4)	56.9 (27.5–124.4)	
Spleen — long axis				< 0.0001
Median (range)	91.9 (59.9–128.0)	117.3 (80.9–203.9)	103.8 (59.9–203.9)	
Spleen — mean HU				< 0.0001
Median (range)	96.5 (41.3–137.1)	84.3 (39.2–137.0)	89.4 (39.2–137.1)	

The group with small spleen volume (SSV) was defined as patients with a spleen volume lower than or equal to 238.0 cm^3^ and the group with large spleen volume (LSV) was defined as patients with a spleen volume higher than 238.0 cm^3^

Medians and ranges were given for continuous variables and percentages for categorial variables

*ML*, acute myeloid leukemia; *t AML*, therapy-associated AML; *s AML*, secondary AML; *FAB*, French-American-British; *ELN*, European Leukemia Net; *Tx*, transplantation date; *CR*, complete remission; *PBSC*, peripheral blood stem cells; *BM*, bone marrow; *CMV*, cytomegalovirus; *D*, donor; *R*, recipient; *MAC*, myeloablative conditioning; *RIC*, reduced intensity conditioning; *NMA*, non-myeloablative conditioning; *SEQ*, sequential conditioning; *TBI*, total body irradiation; *Gy*, gray; *ATG*, anti-thymocyte globulin; *CsA*, cyclosporin A; *MTX*, methotrexat; *MMF*, mycophenolat–mofetil; *GVHD*, graft-versus-host disease; *CD*, cluster of differentiation; *G-CSF*, granulocyte colony-stimulating factor; *HU*, Hounsfield units

### Spleen volume and outcome after HSCT

**Fig. 3:**
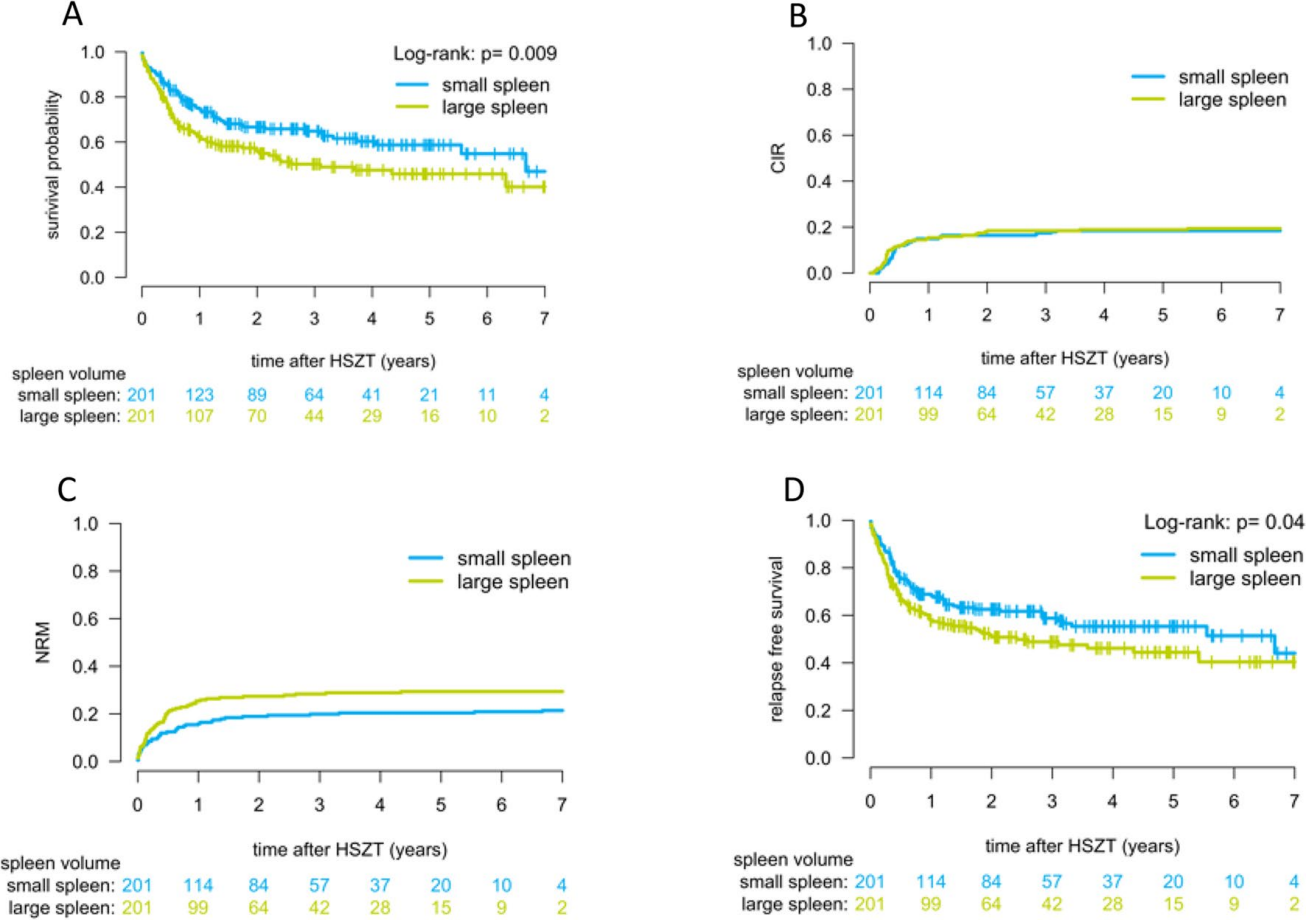
Effect of spleen size on overall survival, relapse, non-relapse mortality, and relapse-free survival. Overall survival (**A**), cumulative incidence of relapse (**B**), non-relapse mortality (**C**), and relapse-free survival (**D**) are displayed for AML patients after HSCT. The group with small spleen volume (SSV) was defined as patients with a spleen volume lower than or equal to 238.0 cm^3^ and the group with large spleen volume (LSV) was defined as patients with a spleen volume higher than 238.0 cm^3^

**Fig. 4:**
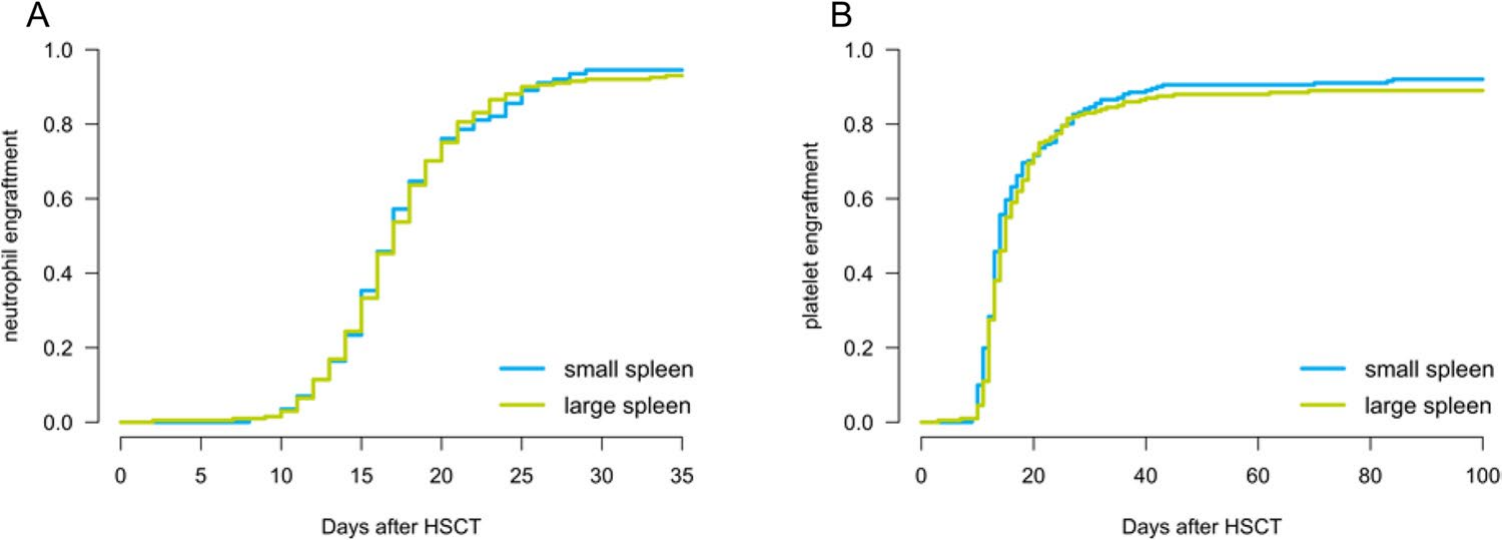
Neutrophil and platelet engraftment after HSCT. Neutrophil (**A**) and platelet (**B**) engraftment kinetics of the large spleen volume (LSV) and small spleen volume (SSV) groups are displayed, with T = 0 at the time of HSCT

Median follow-up was 33.7 months (95% confidence interval [CI], 28.9–37.4 months). LSV was significantly associated with a higher NRM (28.8% vs 20.2% at 2 years; *P* = 0.048; Fig. 3C) and inferior OS after HSCT (55.7% vs 66.6% at 2 years; *P* = 0.009; Fig. 3A). CIR did not differ between both groups (*P* = 0.714; Fig. 3B). We found no differences in time to neutrophil or platelet engraftment between SSV and LSV (Fig. 4). Median time of platelet engraftment was 14 days post HSCT and the median time of neutrophil engraftment was 17 days for both groups. There was no difference between the cumulative incidence of acute and chronic GVHD in both groups (*P* = 0.454 for acute GVHD and* P* = 0.339 for chronic GVHD). Furthermore, the primary cause of death did not significantly differ between both groups (*P* = 0.519), but there was a trend towards more infection-related deaths in the LSV group (41.5% vs 32.9% of all deceased; *P* = 0.329).

In the multivariate regression analyses (Table 2), spleen volume was independently associated with NRM (*P* = 0.035) and OS (*P* = 0.008) after adjusting for age, sex, body length, AML type, HLA match, donor type, remission status, and CMV risk. This was confirmed by a competing risk regression based on Fine and Gray’s proportional subhazards (for NRM and CIR) and Cox proportional hazards (for OS) (Table 2). The adjusted hazard ratio for NRM was 1.55 (95% CI, 1.03–2.34), respectively, for the LSV compared to the SSV group. A co-factor influencing NRM other than spleen volume was remission status (*P* = 0.001). The adjusted hazard ratio for OS was 1.55 (95% CI, 1.12–2.14), respectively, for the LSV compared to the SSV group. Co-factors influencing OS other than spleen volume were age (*P* = 0.018), HLA match (*P* = 0.022), and remission status (*P* = 0.013). Spleen volume was not associated with CIR (*P* = 0.830).

**Table 2:** Multivariate analyses of covariables for overall survival, relapse mortality, and non-relapse mortality

Covariables	OS		CIR		NRM	
	Cox proportional hazards		Fine + Gray		Fine + Gray	
	HR (95%CI)	*P*	HR (95%CI)	*P*	HR (95%CI)	*P*
Age (per 1 year)	1.02 (1.00–1.03)	0.018	1.00 (0.99–1.02)	0.810	1.02 (1.00–1.04)	0.079
Sex						
Male	Ref		Ref		Ref	Ref
Female	0.93 (0.61–1.41)	0.719	0.89 (0.45–1.75)	0.740	1.00 (0.61–1.64)	0.990
Body length (per 1 cm)	1.00 (0.98–1.02)	0.936	1.01 (0.98–1.05)	0.470	0.99 (0.96–1.02)	0.500
AML type						
De novo AML	Ref		Ref		Ref	
t AML	0.88 (0.41–1.93)	0.756	0.45 (0.11–1.90)	0.280	1.44 (0,62–3.36)	0.390
s AML	0.92 (0.62–1.36)	0.669	0.41 (0.20–0.84)	0.015	1.56 (0.98–2.49)	0.064
HLA match						
Matched	Ref		Ref		Ref	
Mismatched	0.63 (0.43–0.94)	0.022	0.92 (0.51–1.68)	0.790	0.70 (0.43–1.12)	0.140
Donor relation						
Related	Ref		Ref		Ref	
Unrelated	0.71 (0.49–1.04)	0.076	0.38 (0.21–0.70)	0.002	1.52 (0.74–1.80)	0.530
Remission status						
CR	Ref		Ref		Ref	
Active disease	1.50 (1.09–2.07)	0.013	0.95 (0.59–1.52)	0.820	1.99 (1.31–3.01)	0.001
CMV risk						
Low	Ref		Ref		Ref	
High	1.35 (0.96–1.88)	0.081	1.35 (0.82–2.23)	0.240	1.19 (0.77–1.83)	0.430
Spleen volume						
≤ 238.0 cm^3^	Ref		Ref		Ref	
> 238.0 cm^3^	1.55 (1.12–2.14)	0.008	1.05 (0.66–1.69)	0.830	1.55 (1.03–2.34)	0.035

*OS*, overall survival; *CIR*, cumulative incidence of relapse; *NRM*, non-relapse mortality; *Ref*, reference; *HR*, hazard ratio; *CI*, confidence interval; *AML*, acute myeloid leukemia; *t AML*, therapy-associated AML; *s AML*, secondary AML; *HLA*, human leukocyte antigen; *CR*, complete remission; *CMV*, cytomegalovirus. Low CMV risk, recipient − / donor − or recipient − /donor + ; high CMV risk, recipient + / donor − or recipient + / donor +

Additional sensitivity analyses were performed using gender-specific cut points for spleen volume (female: 199.1 cm^3^; male: 256.3 cm^3^). Here, spleen volume remained associated with OS for both women and men (*P* = 0.040; Online Resource 3), whereas sex was not associated with OS in the total cohort (*P* = 0.400, Online Resource 4). Analysis of our data, using splenic axis as a predictor for outcome after HSCT in multivariable models, confirmed that spleen volume represents a better indicator for the prediction of outcome than the axis of spleen (Online Resource 1 + 2): with respect to OS, NRM, and CIR, no differences could be seen between groups dichotomized by long or short splenic axis. When body weight was added to the multivariate regression analyses as a co-factor spleen volume remained independently associated with OS (*P* = 0.012).

**Fig. 5:**
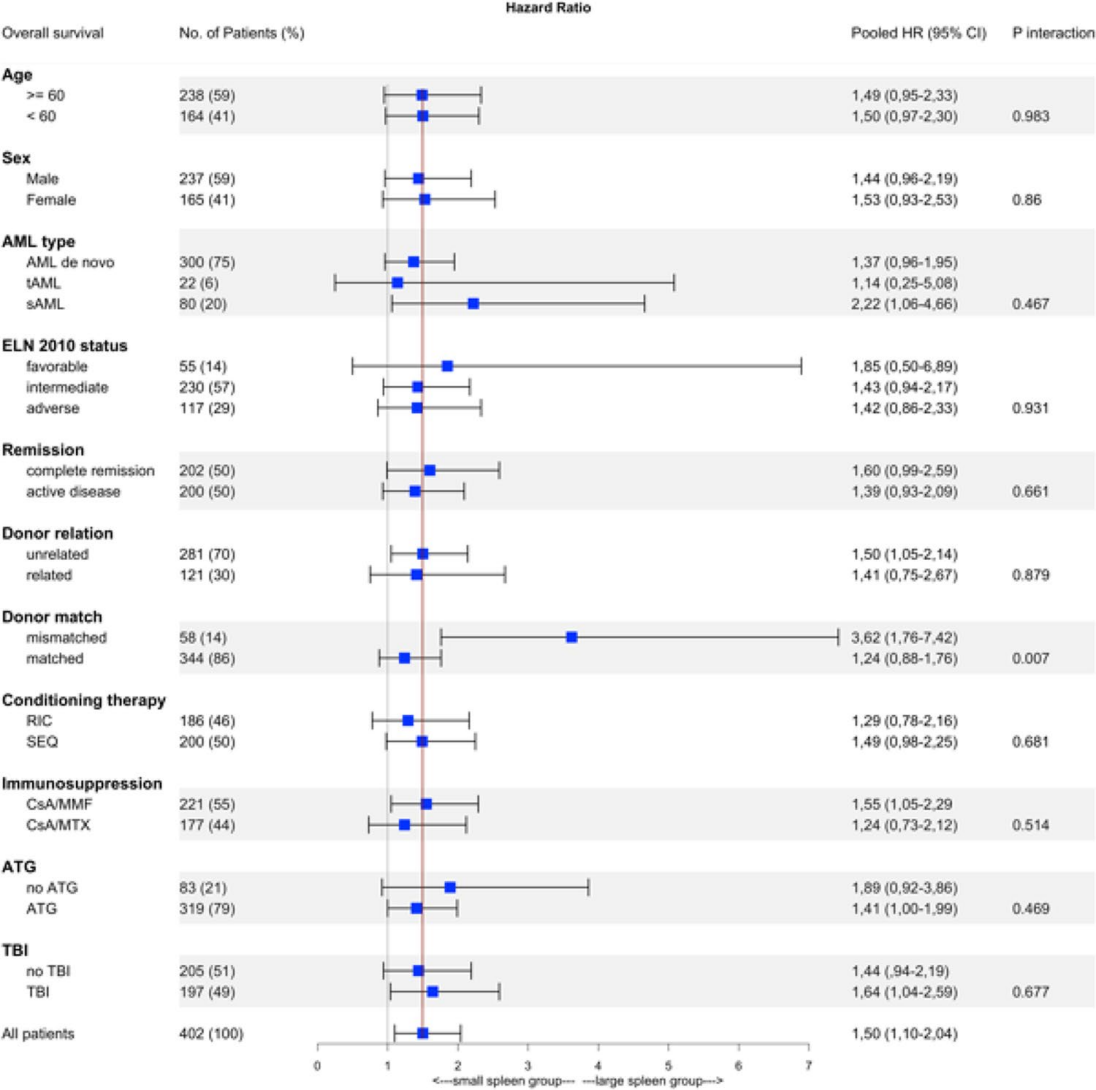
Forest plot representing the association between spleen volume and OS in subgroups. The vertical line represents the hazard ratio of the total cohort. The *P* value was calculated with chi-square test comparing the HRs across subgroups

We found no significant heterogeneity of the associations between spleen volume and OS depending on age, sex, AML type, ELN 2010 status, remission status, donor relation, sex mismatch, conditioning therapy, immunosuppression, and total body irradiation (TBI) (Fig. 5). Significant heterogeneity of associations was observed between spleen volume and OS by HLA match (*P* = 0.007).

## Discussion

Patients with enlarged spleen are reported to have poor and delayed engraftment kinetics that lead to reduced survival following HSCT [9, 10]. However, samples sizes of those studies were small and cohorts heterogenous in terms of diseases. Here, we investigated the association between pretransplant spleen volume, outcome, and hematological recovery in a large cohort of AML patients who underwent HSCT.

Our data implicate that an increased spleen volume is linked to inferior outcome after allogeneic HSCT in AML patients. Two years after HSCT, OS was 66.6% in the SSV group vs. 55.7% in the LSV group. Furthermore, NRM rate was higher for LSV patients, whereas no difference in CIR was found. The association between pretransplant splenomegaly and worse OS and higher NRM described here is consistent with a recent EBMT analysis [16] in patients with myelofibrosis. In line with published literature, we did not find any correlation between spleen volume and relapse rate.

Spleen volume relates to a number of different individual factors such as sex, body stature, and some aspects of the patient’s past medical history reference. As expected, there were more male patients in the LSV group. To eliminate the influence of sex on spleen volume, outcome was, in addition, analyzed for male and female patients separately, revealing that smaller spleen volume was associated with better OS for both women and men. Weight was not taken into account as a co-factor in the multivariate regression analyses due to its instability and correlation with body length, which has the strongest effect on spleen size [17]. However, even when considering body weight in the multivariate regression analyses, spleen volume remained independently associated with OS. Similarly, and as anticipated [17], sex and body length differed between SSV and LSV groups in our cohort. Again, when multivariate analyses were adjusted for these co-factors, spleen volume remained associated with both NRM and OS. Importantly, a history of myeloproliferative neoplasms (e.g., myelofibrosis) could be ruled out as a co-factor influencing our results: myelofibrosis can lead to secondary AML, which has not been described more often in the LSV group. Furthermore, after adding AML type (de novo vs. secondary AML) to the multivariate regression analyses, spleen volume remained independently associated with OS and NRM. In summary, we identified spleen volume as an independent risk factor for NRM.

In patients with myelofibrosis the spleen volume has been shown to represent a better indicator for the prediction of outcome than axis length of spleen [18]. Indeed, additional analysis of our data, using splenic axis as a predictor for outcome after HSCT in multivariable models, confirmed this finding: with respect to OS, NRM, and CIR, no differences could be seen between groups dichotomized by long or short splenic axis.

In contrast to Shimomura et al. [9], there was no difference in time to engraftment between SSV and LSV groups of our cohort. In addition, the proportion of patients in the LSV group that were treated with G-CSF was not different from the SSV group. Furthermore, we found no association between larger spleens and increased incidence of GVHD as other authors did [19], nor was GVHD as the primary cause of death increased in those patients. The effect of spleen volume on NRM remained after multivariable adjustment for stage of disease and GVHD rate. In the present study the adverse outcome was mainly caused by an increased NRM.

The pathophysiological mechanism behind the effect of an enlarged spleen on mortality rate remains unclear. A correlation between late hematological recovery and splenomegaly leading to higher NRM has been described before [16]. However, in our AML cohort, patients with an enlarged spleen did not have delayed engraftment. However, there was a trend towards more infection-related deaths. A possible pathophysiological mechanism could be an association between splenomegaly and a suppressed immune system, resulting in more severe post-transplant infections affecting the OS.

To our knowledge the present cohort is the largest study on the impact of spleen volume on outcome of AML patients after HSCT. Importantly, the cohort studied here is homogenous with only AML patients being included in the study. Limitations of this study are its retrospective, single-center study design potentially causing patient bias. While all patients were diagnosed with AML, we cannot rule out that some patients might have had a myelodysplastic syndrome/ myeloproliferative neoplasm (MDS/MPN) overlap syndrome which can be the cause for larger spleen volume. Furthermore, spleen volume can only be a surrogate parameter because the influence of transfusions and therapeutic agents on spleen volume cannot be excluded.

Our results highlight the importance of considering spleen volume as a measure to assess the outcome of AML patients after HSCT in the future. However, before reducing spleen size (e.g., by radiotherapy) can be considered a therapeutic option to positively influence outcome in posttransplant AML, the pathophysiological mechanisms underlying the observed associations need to be clarified.

In conclusion, our study identifies spleen volume as an independent risk factor for adverse outcome in AML patients undergoing allogeneic HSCT, mainly due to increased rates of NRM, while engraftment kinetics were not affected.

## Supplementary information

Below is the link to the electronic supplementary material.

Supplementary file1 (DOCX 1079 KB)

## Data Availability

Data not available due to ethical restrictions.

## References

[1] Gooley TA, Chien JW, Pergam SA (2010). Reduced mortality after allogeneic hematopoietic-cell transplantation. N Engl J Med.

[2] Boeckh M (2008). The challenge of respiratory virus infections in hematopoietic cell transplant recipients. Br J Haematol.

[3] Kurosawa S, Yakushijin K, Yamaguchi T (2013). Changes in incidence and causes of non-relapse mortality after allogeneic hematopoietic cell transplantation in patients with acute leukemia/myelodysplastic syndrome: an analysis of the Japan Transplant Outcome Registry. Bone Marrow Transplant.

[4] Kurosawa S, Yakushijin K, Yamaguchi T (2013). Recent decrease in non-relapse mortality due to GVHD and infection after allogeneic hematopoietic cell transplantation in non-remission acute leukemia. Bone Marrow Transplant.

[5] Gratwohl A, Brand R, Frassoni F (2005). Cause of death after allogeneic haematopoietic stem cell transplantation (HSCT) in early leukaemias: an EBMT analysis of lethal infectious complications and changes over calendar time. Bone Marrow Transplant.

[6] Horan JT, Logan BR, Agovi-Johnson M-A (2011). Reducing the risk for transplantation-related mortality after allogeneic hematopoietic cell transplantation: how much progress has been made?. J Clin Oncol.

[7] Bacigalupo A, Soraru M, Dominietto A (2010). Allogeneic hemopoietic SCT for patients with primary myelofibrosis: a predictive transplant score based on transfusion requirement, spleen size and donor type. Bone Marrow Transplant.

[8] Gergis U, Kuriakose E, Shore T (2016). Allogeneic transplantation for patients with advanced myelofibrosis: splenomegaly and high serum LDH are adverse risk factors for successful engraftment. Clin Lymphoma Myeloma Leuk.

[9] Shimomura Y, Hara M, Katoh D (2018). Enlarged spleen is associated with low neutrophil and platelet engraftment rates and poor survival after allogeneic stem cell transplantation in patients with acute myeloid leukemia and myelodysplastic syndrome. Ann Hematol.

[10] Akpek G, Pasquini MC, Logan B (2013). Effects of spleen status on early outcomes after hematopoietic cell transplantation. Bone Marrow Transplant.

[11] Harris AC, Young R, Devine S (2016). International, multicenter standardization of acute graft-versus-host disease clinical data collection: a report from the Mount Sinai Acute GVHD International Consortium. Biol Blood Marrow Transplant.

[12] Glucksberg H, Storb R, Fefer A (1974). Clinical manifestations of graft-versus-host disease in human recipients of marrow from HL-A-matched sibling donors. Transplantation.

[13] Filipovich AH, Weisdorf D, Pavletic S (2005). National Institutes of Health consensus development project on criteria for clinical trials in chronic graft-versus-host disease: I. Diagnosis and staging working group report. Biol Blood Marrow Transplant.

[14] Jagasia MH, Greinix HT, Arora M (2015). National Institutes of Health Consensus Development Project on Criteria for Clinical Trials in Chronic Graft-versus-Host Disease: I The 2014 Diagnosis and Staging Working Group report. Biol Blood Marrow Transplant.

[15] Copelan E, Casper JT, Carter SL (2007). A scheme for defining cause of death and its application in the T cell depletion trial. Biol Blood Marrow Transplant.

[16] Polverelli N, Mauff K, Kröger N (2021). Impact of spleen size and splenectomy on outcomes of allogeneic hematopoietic cell transplantation for myelofibrosis: a retrospective analysis by the chronic malignancies working party on behalf of European society for blood and marrow transplantation (EBMT). Am J Hematol.

[17] Chow KU, Luxembourg B, Seifried E (2016). Spleen size is significantly influenced by body height and sex: establishment of normal values for spleen size at US with a cohort of 1200 healthy individuals. Radiology.

[18] Song M-K, Chung J-S, Lim S-N (2016). Usefulness of spleen volume measured by computed tomography for predicting clinical outcome in primary myelofibrosis. Int J Hematol.

[19] Boström L, Ringdén O (1992). No association between splenomegaly and acute graft-vs.-host disease in humans after allogeneic bone marrow transplantation. Transplant Proc.

