# Supplementary material for: Pretransplant spleen volume and outcome after hematopoietic stem cell transplantation (HSCT) in patients with acute myeloid leukemia (AML)
Source: Ann Hematol. 2023 Jul 10;102(9):2543–53. doi: 10.1007/s00277-023-05353-9 (PMC10444671; doi:10.1007/s00277-023-05353-9)
Supplement: Supplementary file 1 — Supplementary file1 (DOCX 1079 KB) [file 277_2023_5353_MOESM1_ESM.docx]

**Pretransplant spleen volume and outcome after hematopoietic stem cell transplantation (HSCT) in patients with acute myeloid leukemia (AML)**

**Annals of Hematology**

Alexander Pohlmann^1*^, Eva Bentgens^1*^, Christoph Schülke^2^, David Kuron^3^, Christian Reicherts^1^, Julia Marx^1^, Linus Angenendt^1^, Jan-Henrik Mikesch^1^, Georg Lenz^1^, Matthias Stelljes^1^, Christoph Schliemann^1^

^1^*Department of Medicine A, University Hospital Münster, Münster, Germany*

^2^*Department of Clinical Radiology, University Hospital Münster, Münster, Germany*

*^3^ Department of Medicine II, University Hospital Schleswig-Holstein, 24105 Kiel, Germany*

* *These authors contributed equally to this work*

**Corresponding author**

Christoph Schliemann, M.D., Department of Medicine A, University Hospital Münster, Münster, Germany; email: Christoph.Schliemann@ukmuenster.de

| OS | NRM | CIR |
| --- | --- | --- |
| 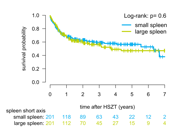 | 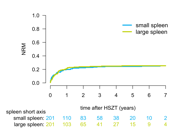 | 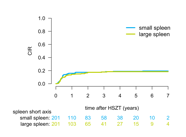 |
| Online Resource 1 Effect of splenic short axis on OS, NRM and CIR | | |

There were no differences concerning OS (*P* = 0.600), NRM (*P* = 0.986) and CIR (*P* = 0.786) between SSV and LSV groups.

| OS | NRM | CIR |
| --- | --- | --- |
| 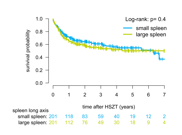 | 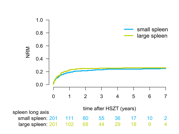 | 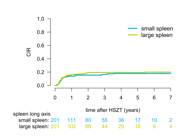 |
| Online Resource 2 Effect of splenic long axis on OS, NRM and CIR | | |

There were no differences concerning OS (*P* = 0.400), NRM (*P* = 0.781) and CIR (*P* = 0.598) between SSV and LSV groups.

| OS | NRM | CIR |
| --- | --- | --- |
| 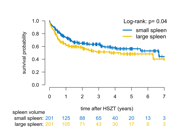 | 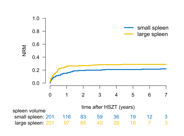 | 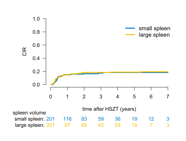 |
| Online Resource 3 Effect of spleen volume on OS, NRM and CIR with groups for both sexes divided by median spleen volume (female 199.1 cm^3^, male 256.3 cm^3^) | | |

There were no difference concerning NRM (*P* = 0.067) and CIR (*P* = 0.685) between the SSV and LSV groups, but a difference in OS could be seen (*P* = 0.040).

| 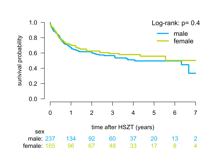 |
| --- |
| Online Resource 4 Effect of sex on OS |
